# Hepatocellular carcinoma may display elevated nestin expression in endothelial cells: experimental study: O carcinoma hepatocelular pode expressar nestina fortemente em células endoteliais: estudo experimental

**DOI:** 10.1590/1516-3180.2014.8670910

**Published:** 2014-11-28

**Authors:** Adriano Barreto Nogueira, Ariel Barreto Nogueira, Anderson Lino Costa, Fabiana Roberto Lima, Sheila Aparecida Siqueira, Manoel Jacobsen Teixeira

**Affiliations:** I MD. Attending Physician, Division of Clinical Neurosurgery, and Researcher, Laboratory of Experimental Surgery, Hospital das Clínicas, Faculdade de Medicina da Universidade de São Paulo (HC/FMUSP), São Paulo, Brazil.; II MD. Pathologist and Radiology Resident, Institute of Radiology, Hospital das Clínicas, Faculdade de Medicina da Universidade de São Paulo (HC/FMUSP), São Paulo, Brazil.; III MD. Attending Physician, Department of Pathology, Hospital das Clínicas, Faculdade de Medicina da Universidade de São Paulo (HC/FMUSP), São Paulo, Brazil.; IV MD, PhD. Director, Department of Pathology, Hospital das Clínicas, Faculdade de Medicina da Universidade de São Paulo (HC/FMUSP), São Paulo, Brazil.; V MD, PhD. Professor of Neurosurgery, Department of Neurology, and Head of Department, Division of Clinical Neurosurgery, Faculdade de Medicina da Universidade de São Paulo (HC/FMUSP), São Paulo, Brazil.

**Keywords:** Nestin, Endothelial cells, Neovascularization, pathologic, Carcinoma, hepatocellular, Immunohistochemistry

## Abstract

**CONTEXT AND OBJECTIVE:**

Nestin, a class VI intermediate filament protein, is highly expressed in the portal mesenchyme and sinusoidal endothelium of the human fetal liver, but scarcely expressed in adult portal vessel endothelium. During experimental liver regeneration, an increased number of nestin-positive parenchymal cells have been observed in the zone adjacent to the Hering canals. These parenchymal cells are regarded as hepatic stem cells or hepatoblasts, which may be involved in hepatocellular carcinogenesis. In the light of recent reports describing nestin-positive parenchymal cells in hepatocellular carcinoma, we aimed to use this tumor type as a positive control for immunohistochemical detection of nestin.

**DESIGN AND SETTING:**

Experimental study conducted at a university hospital.

**METHODS:**

Hepatocellular carcinoma sections from one case were analyzed for nestin expression by immunohistochemistry using confocal microscopy.

**RESULTS:**

Surprisingly, a conspicuous pattern resembling liver sinusoid-like cytoarchitecture was observed upon nestin staining of endothelial cells.

**CONCLUSIONS:**

This pattern has not been previously described. The preliminary results shown here suggest that nestin-positive endothelial cells are located in niches of immature or proliferative cells. Moreover, nestin expression in endothelial cells of hepatocellular carcinoma enhances the role of angiogenesis in this tumor type, although the prevalence of this immunohistopathological pattern remains to be determined. Finally, hepatocellular carcinoma is an effective positive control for nestin staining in fluorescent immunohistochemistry.

## RESUMO

**CONTEXTO E OBJETIVO:**

Nestina, proteína de filamento intermediário de classe VI, é altamente expressa no mesênquima portal e endotélio sinusoidal do fígado fetal humano, mas pouco expressa no endotélio de vasos portais no adulto. Além disso, durante a regeneração hepática experimental, um número crescente de células parenquimatosas nestina-positivas é observado na zona adjacente aos canais de Hering. Essas células parenquimatosas são consideradas células-tronco hepáticas ou hepatoblastos, que podem estar envolvidas na origem do carcinoma hepatocelular. Em função de relatos recentes descrevendo células parenquimatosas nestina-positivas no carcinoma hepatocelular, objetivamos usar este tipo tumoral como controle positivo para a detecção imunoistoquímica da nestina.

**TIPO DE ESTUDO E LOCAL:**

Estudo experimental realizado em hospital universitário.

**MÉTODOS:**

Cortes de um caso de carcinoma hepatocelular foram analisados em relação à expressão imunoistoquímica da nestina por microscopia confocal.

**RESULTADOS:**

Surpreendentemente, um padrão nítido com citoarquitetura semelhante a sinusoides hepáticos foi observado pela marcação por nestina em células endoteliais.

**CONCLUSÕES:**

Este padrão não havia sido descrito previamente. Os resultados preliminares mostrados aqui sugerem que células endoteliais nestina-positivas estão localizadas em nichos de células imaturas ou em proliferação. Além disto, a expressão de nestina em células endoteliais de carcinoma hepatocelular reforça o papel da angiogênese neste tipo tumoral, embora a prevalência deste padrão imuno-histopatológico precise ser determinada. Finalmente, o carcinoma hepatocelular é um controle positivo efetivo para a marcação para nestina em imunoistoquímica fluorescente.

## INTRODUCTION

The search for a specific marker of neural stem cells during brain development has led to identification and isolation of the gene encoding nestin,^1^ a class VI intermediate filament protein. Since then, nestin staining has been described in the embryogenesis of tissues originating from all three germ layers as well as in relation to regeneration, tumorigenesis and physiological conditions in adult mammals.^2^^,^^3^ Therefore, nestin has been detected under different conditions, in a wide variety of tissues and at a broad range of intensities, which has hindered identification of its actual function.

Nestin is expressed in the portal mesenchyme and the sinusoidal endothelium of the liver during development, and in a few endothelial cells of the portal vessels in adult individuals.^4^ Moreover, hepatic stem cells, which express nestin, have been characterized in cultured adult human liver cells *in vitro*.^5^

However, controversy continues regarding the phenotypic and histological features of human hepatic stem cells.^5^^,^^6^ Hepatocyte regeneration via hepatic stem cells occurs primarily within the topography of the Hering canals (the distal structure of biliary trees), towards the central vein. According to one study, the Hering canal cells are hepatic stem cells and are responsible for the ductal reaction during human liver regeneration and for the genesis of cholangiocytes and hepatocytes. Hepatoblasts, which line the Hering canals, are transit-amplifying cells and give rise to hepatocytes.^6^ Another study has indicated that hepatic stem cells are more lineage-restricted *in vivo*,^5^ similar to the hepatoblasts described above. Importantly, we are not aware of any studies showing nestin expression in tissue slices of human liver during regeneration. However, nestin has been shown to be present in a subset of oval cells, which are murine progenitor cells spawned during liver regeneration.^7^ Taken together, these data suggest that hepatic stem cells may be classified as parenchymal cells.

Hepatic stem cells may give rise to hepatocellular carcinoma.^8^^,^^9^^,^^10^ Accordingly, clusters of nestin-positive parenchymal cells have been observed in hepatocellular carcinoma slices.^10^ Moreover, it has been suggested that hepatocellular carcinoma is a positive control for nestin expression, because of the staining of parenchymal cells, according to the data sheet from a commercially available anti-nestin primary antibody.^11^

## OBJECTIVES

We performed nestin staining in hepatocellular carcinoma slices with the aim of identifying a reliable positive control for immunohistochemical detection of this protein,^12^ and report this preliminary result in order to discuss the role of nestin-expressing endothelial cells, particularly in hepatocellular carcinoma angiogenesis.

## METHODS

This study was carried out after receiving approval from the Ethics Committee for Research Project Analysis (CAPPesq) of the Clinics Hospital, University of São Paulo Medical School (Hospital das Clínicas, Faculdade de Medicina da Universidade de São Paulo, HC/FMUSP) (research protocol no. 013/05).

The Department of Pathology of HC/FMUSP provided us with a paraffin block of embedded hepatocellular carcinoma containing three fragments from the same clinical case, and a glioblastoma sample for a complementary experiment, to observe nestin staining on another tissue. The hepatocellular carcinoma block was cut into 5-µm slices, which were mounted on silanized slides. Hematoxylin-eosin staining was performed on a parallel slide, in accordance with standard protocols.

Immunohistochemistry was performed on the hepatocellular carcinoma slices based on an immunofluorescence protocol that we had developed previously.^12^ In summary, the slices were deparaffinized in xylene and rehydrated in ethanol. To diminish auto-fluorescence, the slides were immersed in 1% Sudan Black B (Sigma-Aldrich, Saint Louis, MO, USA) diluted into 70% ethanol, for 30 minutes. After washing in phosphate-buffered saline, the slides were separated into four groups, according to the solution used for microwave antigen retrieval and the type of anti-nestin primary antibody used for detection: 1) ethylenediaminetetraacetic acid buffer (1 mM, pH 8.0) and rabbit polyclonal antibody (catalog no. ab93666, Abcam, Cambridge, MA, USA); 2) sodium citrate buffer (10 mM, pH 6.0) and rabbit polyclonal antibody (as in the preceding group); 3) sodium citrate buffer and mouse monoclonal antibody (catalog no. ab22035, Abcam); and 4) sodium citrate buffer and mouse monoclonal antibody from a second manufacturer (catalog no. MAB5326, Millipore, Billerica, MA, USA). After heating in a microwave, the slides were kept at room temperature for 30 min, washed in phosphate-buffered saline (PBS) containing 0.2% Triton X-100 (PBS-Triton) and then incubated in a blocking solution (1% bovine serum albumin and goat serum (1:20) diluted into PBS-Triton) for 1 h. The anti-nestin primary antibody was then applied at three different concentrations (1:50, 1:100 and 1:200) to all slide groups. For the negative control, we omitted the anti-nestin antibody on one of the slides in each group. All slides were kept in a humidified chamber at 4 °C for 48 hours.

The slides were then washed in PBS-Triton and incubated with biotinylated secondary antibodies (goat anti-rabbit immunoglobulin G (IgG)-biotin antibody, 1:1,000, catalog no. B8895, Sigma-Aldrich, Saint Louis, MO, USA; goat anti-mouse IgG-biotin antibody, 1:200, catalog no. B7151, Sigma-Aldrich) for 90 minutes. After another wash, streptavidin conjugated with a fluorescent probe (streptavidin, Alexa Fluor 488 conjugate, 1:1,000, catalog no. S-32354, Life Technologies, Carlsbad, CA, USA) was applied for 1 h, and the slides were washed again. 4’,6-diamidino-2-phenylindole (DAPI; 1:1,000 w/v, catalog no. 46190, ThermoScientific, Rockford, IL, USA) was used to stain the nuclei. A final wash was carried out, an anti-fading solution (Vectashield, catalog no. H1000, Vector Laboratories, Burlingame, CA, USA) was applied and coverslips were used to cover the hepatocellular carcinoma slices.

The hepatocellular carcinoma slides were analyzed using a Zeiss 510 UV META microscope (Carl Zeiss MicroImaging, Thornwood, NY, USA), and the images were saved and edited using the LSM 510 META software (Zeiss MicroImaging). To generate a panel that included a negative control and a representative result, the images were edited further using Photoshop CS5 Extended software (Adobe Systems Incorporated, San Jose, CA, USA).

To illustrate the features of nestin staining on another tissue, we used a glioblastoma sample and another anti-nestin primary antibody (rabbit polyclonal antibody, 1:5,000, catalog no. AB5922, Millipore). In this case, a labeled streptavidin biotin kit was used, as recommended by the manufacturer (Dako LSAB+ System-HRP, catalog no. K0690, Dako Denmark, Glostrup, Denmark). Bright-field microscopy was performed using a Nikon Optiphot-2 microscope (Nikon, Tokyo, Japan) to analyze the nestin staining pattern in the glioblastoma and the hematoxylin-eosin staining of the hepatocellular carcinoma sample. The images were captured using a CoolSNAP Pro *CF* color digital camera (Media Cybernetics, Inc., Bethesda, MD, USA), saved using the Image-Pro Plus software (Media Cybernetics) and edited using the Photoshop CS5 Extended software (Adobe).

## RESULTS

**Figure 1.**
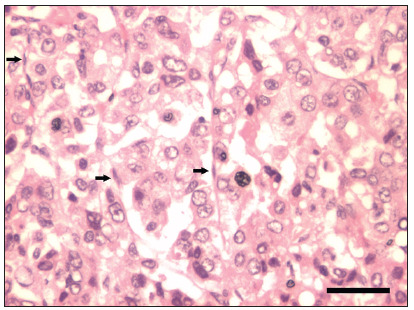
Hematoxylin-eosin staining of hepatocellular carcinoma. Endothelial cells, in increased numbers in comparison with those of a normal liver, were identified by their thin and elongated morphology (arrows). These cells were gathered in lines around tumor parenchymal cells, which usually showed cuboid morphology. Scale bar: 50 µm.

**Figure 2.**
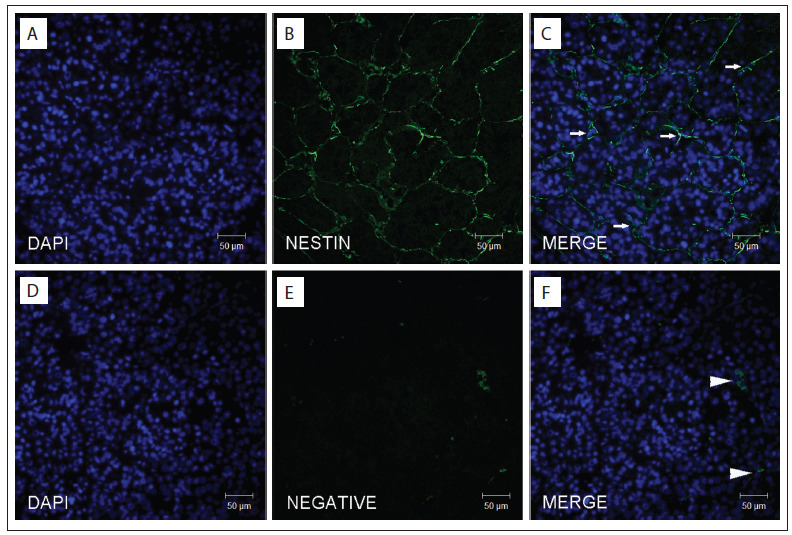
Nestin-positive endothelial cells in hepatocellular carcinoma. The upper row depicts nuclear staining with DAPI (A), nestin staining in endothelial cells that form a liver sinusoid-like structure (B) and a merged image (C). The intense nestin staining in most endothelial cells (arrows), the typical morphology of these cells and the high number of tumor parenchymal cells between the endothelial cell lines contributed to the resulting net image, which was readily detected in tumor nodules via confocal microscopy. The lower row shows the negative control, as analyzed by means of filters for DAPI (D) and Alexa Fluor 488 (E), and a merged image (F). Note that the auto-fluorescence of the red blood cells could not be completely eliminated (arrowheads). The background image, shown in E, can be used for comparison with the nestin staining shown in B. Scale bar: 50 µm.

All three fragments of the hepatocellular carcinoma sample harbored nodules of various sizes, and their histological features are shown in Figure 1. These nodules were surrounded by fibrosis, in which some blood vessels were detected. Major auto-fluorescence was prevalent only in the red blood cells, yet identification of true staining was not significantly hampered because the red blood cells were localized primarily to blood vessels within the fibrotic areas. In addition, the presence of a large number of endothelial cells, characterized by their thin and elongated shape and the overall appearance of a liver sinusoid-like structure, allowed confident identification of nestin staining. An illustrative example is depicted in Figure 2, for which the polyclonal antibody and the sodium citrate buffer were used. The best image was achieved with the 1:100 dilution of the primary antibody for all groups compared with the 1:50 or 1:200 dilutions. The type of anti-nestin primary antibody and the buffer solution for antigen retrieval did not influence the results. Another factor contributing to effective identification of nestin-positive endothelial cells was the scarce markings of other cells (e.g. tumor parenchymal cells). Finally, the nestin staining did not always form a continuous net pattern; the overall result was a spectrum, in which nodules with a range of discontinuous figures were detected, possibly reflecting different degrees of angiogenesis.

**Figure 3.**
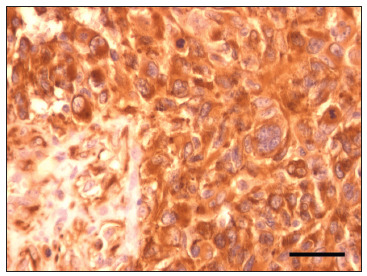
Nestin staining of the glioblastoma sample. Nestin staining with 3,3’-diaminobenzidine as a chromogen demonstrated that it is highly expressed in glioblastoma. This staining could easily be distinguished if the immunohistochemistry reaction was revealed through the immunoenzymatic reaction but not through the immunofluorescence method. Scale bar: 50 µm.

Immunohistochemistry on the glioblastoma sample revealed a diffuse nestin staining pattern (Figure 3). However, when fluorescent probes were used, the intense auto-fluorescence of this type of tumor (data not shown) hindered confident identification of cells stained for nestin. Therefore, hepatocellular carcinoma is a more suitable option for a nestin-positive control than glioblastoma, when using fluorescent immunohistochemistry.

## DISCUSSION

Nestin-positive endothelial cells are considered to be a marker of newly formed blood vessels.^13^ As in highly angiogenic tumors, nestin is expressed in endothelial cells of many organs during development or regeneration, in both humans^3^ and experimental models.^14^ Rat pancreatic endothelial cells express nestin during pancreatic regeneration but not in the quiescent state.^14^ Moreover, nestin-positive endothelial cells and nestin-negative endothelial cells may be found in the same rat brain: for example, in glioblastoma and in normal adjacent tissue, respectively.^13^

In contrast, there is evidence suggesting that nestin-positive endothelial cells may not be related to newly formed blood vessels,^15^^,^^16^ similarly to the conditions under which nestin is detected in non-endothelial mature cells.^3^ In the human pancreas, nestin-positive endothelial cells have been found in both endocrine and exocrine tissues, in numbers that exceed the turnover of endothelial cells.^15^ Likewise, a study on the coupling of angiogenesis and neurogenesis in the adult rat brain showed that nestin-positive endothelial cells were found in mature capillaries.^16^ Moreover, we found nestin-positive endothelial cells in non-pathological adult human brain tissue.^12^ Nonetheless, these cells may have a paracrine effect in tissue-specific progenitor cells during regeneration. Indeed, in the human exocrine pancreas, nestin-positive endothelial cells are adjacent to regions in the ductal epithelium that harbor putative islet progenitor cells.^15^ Furthermore, in our previous study,^12^ nestin upregulation in brain endothelial cells could have occurred due to the occasional hypoxia that occurs during the agonal period, in a process associated with neuroblast migration during injury-induced neurogenesis.^17^ Therefore, one interesting hypothesis to be tested is whether nestin-positive endothelial cells indicate a niche with regenerative potential.

Hepatic stem cell features have been described in a subgroup of hepatocellular carcinomas that are clinically characterized by a poor outcome.^8^^,^^9^^,^^10^ In one study, high serum levels of neural cell adhesion molecule, which is also a hepatic stem cell marker, were detected in a group of hepatocellular carcinoma patients,^9^ and tumor specimens from a smaller proportion of these cases harbored clusters of cells that were positive for the neural cell adhesion molecule. Furthermore, some hepatocellular carcinoma cases showed a gene expression pattern comparable to that presented by rat embryo hepatoblasts, as analyzed by means of a DNA microarray.^8^ Finally, using the quantitative real-time polymerase chain reaction and a tissue microarray, Yang et al.^10^ demonstrated that cases with high expression of hepatic stem cell markers were correlated with increased angiogenesis and poor prognosis; in addition, nestin expression was detected in parenchymal cells. Our finding of significant nestin expression in hepatocellular carcinoma endothelial cells may clarify some aspects of the role of nestin in the proliferation and angiogenesis of this tumor type.

The presence of nestin-positive endothelial cells in hepatocellular carcinoma suggests that there is a remarkable degree of angiogenesis. However, the lineage of these endothelial cells is unknown. First, it is possible that these cells are sinusoidal endothelial cells that switched from a quiescent to a proliferative state (angiogenic switch).^18^ However, some hepatocellular carcinoma endothelial cells may arise from circulating endothelial progenitor cells.^18^ Additional studies are needed to determine whether other types of nestin-expressing cells could give rise to hepatocellular carcinoma endothelial cells. In this manner, tumor stem cells could generate hepatocellular carcinoma endothelial cells, in the same way as has recently been demonstrated for glioblastoma.^19^^,^^20^ Finally, we speculate that stellate cells (pericytes),^21^ which are known to modulate tumor angiogenesis,^18^ could also give rise to tumor endothelial cells under the influence of vascular endothelial growth factor. This growth factor is present at high levels in hepatocellular carcinoma, and pericytes show niche-dependent pluripotency in other organs.^22^

Regardless of the role that nestin expression plays in endothelial cells, the conspicuous pattern shown here makes hepatocellular carcinoma samples a useful alternative positive control for nestin for the following reasons. First, this pattern is particularly valuable for investigators engaged in analyzing fluorescent images. Second, laboratory animal tissue (e.g. murine embryos) may be replaced by biopsy samples,^23^ which is particularly important if the researcher is using an anti-nestin primary antibody that reacts exclusively with human tissue. Third, use of hepatocellular carcinoma samples may avoid the need for the procurement of human fetal brain tissue.^24^

One caveat of our study is that we only used one hepatocellular carcinoma case, yet the lack of information regarding a subject with such relevance and the results presented here warrant this report. For instance, although hepatocellular carcinoma is the fifth most frequent tumor type, its prognosis is highly heterogeneous.^8^^,^^9^^,^^10^ Additionally, nestin is a broadly used molecule in regenerative medicine and in oncology research,^3^ yet a PubMed search (as of April 2, 2014) with the keywords “hepatocellular carcinoma” and “nestin” resulted in only three articles. Finally, the nestin pattern shown here, i.e. hepatocellular carcinoma with elevated nestin expression in endothelial cells, may motivate additional studies to determine its prevalence and prognostic implication.

## CONCLUSIONS

Our findings reinforce the hypothesis that nestin-positive endothelial cells are found in niches of immature or proliferative cells. Likewise, the results are in agreement with the role of angiogenesis in hepatocellular carcinoma. Additional studies are required to determine the prevalence and prognostic value of the immunohistopathological pattern shown here. Finally, hepatocellular carcinoma samples may serve as a useful positive control for nestin in immunohistochemistry experiments.
